# Enhancing an International Perspective in Public Health Teaching through Formalized University Partnerships

**DOI:** 10.3389/fpubh.2017.00036

**Published:** 2017-03-09

**Authors:** Patrick Brzoska, Seval Akgün, Bassey E. Antia, K. R. Thankappan, Kesavan Rajasekharan Nayar, Oliver Razum

**Affiliations:** ^1^Epidemiology Unit, Faculty of Behavioral and Social Sciences, Institute of Sociology, Chemnitz University of Technology, Chemnitz, Germany; ^2^Department of Epidemiology and International Public Health, School of Public Health, Bielefeld University, Bielefeld, Germany; ^3^Department of Public Health, Faculty of Medicine, Başkent University, Ankara, Turkey; ^4^Department of Linguistics, University of the Western Cape, Bellville, South Africa; ^5^Sree Chitra Tirunal Institute for Medical Sciences and Technology, Achutha Menon Centre for Health Science Studies, Trivandrum, India; ^6^Santhigiri Social Research Institute, Trivandrum, India; ^7^Global Institute of Public Health, Trivandrum, India

**Keywords:** International Public Health, university partnership, developing countries, virtual classroom, collaboration

## Abstract

Teaching in the field of public health needs to employ a global perspective to account for the fact that public health problems and solutions have global determinants and implications as well. International university partnerships can promote such a perspective through the strengthening of cooperation, exchange, and communication between academic institutions across national boundaries. As an example for such an academic network in the field of public health, we introduce the International Public Health Partnership—a collaboration between a university in Germany and universities in India, Turkey, and Nigeria. Formed in 2005, it facilitated the exchange of information, fostered discussion about the transferability of public health concepts, contributed to the structural development of the universities involved, and promoted an intercultural dialog through a combination of local and distance learning activities. Although well accepted by students and staff, different obstacles were encountered; these included limited external funding, scarce own financial, time and personnel resources, and diverging regulations and structures of degree programs at the partnership sites. In the present article, we share several lessons that we learned during our joint collaboration and provide recommendations for other universities that are involved in partnerships with institutions of higher education or are interested to initiate such collaborations.

## Introduction

Public health challenges, such as epidemics of communicable and non-communicable diseases as well as questions of inequality, of health care reform and of financing have global dimensions and determinants. Solutions to public health problems, therefore, also require a global perspective and international cooperation in teaching students who are the future specialists in these fields (1, 2).

Health inequalities would be an example of one such public health challenge. Even though at different absolute levels, health inequalities exist worldwide, both in industrialized and in developing countries (3). To a significant degree, they are caused by economic and social factors affecting health through multiple pathways on the psychosocial, behavioral, and material level (4). These economic and social determinants comprise a differential distribution of resources, such as income, education, employment and housing, and environmental factors, such as climate and air, water, and food quality (5, 6). In addition, access to health-care services and quality of care vary across socioeconomic groups, adding to differentials in morbidity and mortality. Also, risk factors, such as tobacco use, tend to have a higher prevalence among poor population groups, while the uptake of preventive interventions against such risk factors is often higher among the well-off than the poor, leading to a further increase in health inequalities (7).

Avoidable or remediable health inequalities between groups of people, whether those groups are defined socially, economically, demographically, or geographically, are unfair and should be reduced (unfair inequalities are often termed inequities). Maintaining and promoting the health of populations and reducing health inequalities are key goals of all national health systems (7). Because societies differ in their approaches to defining, investigating, and tackling inequalities, as well as in their respective success in this socially important endeavor, researchers and policymakers can learn from their experiences (8). A global approach, incorporating the perspective of both developing and developed countries, thus, is indispensable in teaching inequalities in health. The same is true for many other aspects of public health and the health sciences (9, 10).

For several reasons, however, an international perspective in teaching is often limited: existing public or community health courses often focus on national or regional problems than on international aspects of public health (11). Although degree programs that contain coursework in global and international health can convey an international perspective, these programs can benefit strongly from the direct exchange of public health students and lecturers between developing and developed countries with differing cultural and political background. This exchange, however, is often constrained by limited financial and personnel resources. Current approaches to electronic distance learning that could potentially reduce these barriers do not always succeed in overcoming the reservations of lecturers, e.g., with respect to perceived time commitment and inadequate instructor training. They may also make it difficult to maintain the interest and motivation of students (12).

Formalized international university partnerships that foster exchange and cooperation can help tackle these challenges by providing a structural and organizational framework for respective activities (13, 14). They can also be a competitive edge for universities in terms of international visibility and attractiveness for international students and researchers. Such collaborations, however, make huge demands on financial resources, individual commitment of the persons involved, and available infrastructure. They are also challenged by geographical distance as well as cultural, political, and economic diversity (15, 16). In this article, we introduce the International Public Health Partnership between a university in Germany and universities in India, Turkey, and Nigeria as an example for such an academic collaboration in the field of public health. We illustrate the benefits of international cooperation in public health education and describe the use of different teaching instruments as well as the potential obstacles that need to be overcome in order to achieve a sustainable cooperation. This may provide helpful information for other universities that are involved in partnerships with institutions of higher education or are interested to initiate such collaborations.

## Collaboration on Equal Terms: The International Public Health Partnership

The International Public Health Partnership was founded by the School of Public Health at Bielefeld University, Germany; the Centre of Social Medicine and Community Health at the Jawaharlal Nehru University in New Delhi, India; and the Department of Public Health at the Başkent University in Ankara, Turkey in 2005. The initiative was a response to the increasing need for a global perspective in public health research and practice. Later, the Achutha Menon Centre for Health Science Studies of the Sree Chitra Tirunal Institute for Medical Sciences and Technology in Trivandrum, India, and the Department of Languages and Linguistics at the University of Maiduguri in Maiduguri, Nigeria, joined.

The academic collaboration originated from informal personal and professional contacts and was financially supported for the first 8 years with a total budget of between 25,000€ and 35,000€ per year by the German Academic Exchange Service [*Deutscher Akademischer Austausch Dienst (DAAD)*] through its “Subject-related Partnerships with Institutions of Higher Education in Developing Countries” program (17). Afterward, German–Indian activities received funding through the government program “A New Passage to India,” administered *via* DAAD.

### Rationale and Concept

The overall goal of the International Public Health Partnership was to improve the structural basis of teaching in international public health, to broaden understanding of the multi- and interdisciplinary foundations of scholarship and initiatives in international public health, to facilitate exchange of information, and to initiate discussion around the transferability of public health concepts between the institutes involved. The diverse activities of the partnership aimed to offer a possibility for students and lecturers to experience and jointly analyze international aspects of public health, its ethical implications, the responsibilities of stakeholders, and the effectiveness of policies and strategies to reduce global and local public health problems. Students from Nigeria, India, and Turkey could benefit from a first-hand experience of public health problems and constraints the German health system is facing, thus informing the debate on public health priorities. On the other hand, German students could benefit from a practical exposure to issues in health care in a less-affluent setting, with such an experience serving as an eye-opener and facilitating a better understanding of public health. Additionally, the collaboration sensitized staff and students toward issues of developmental policy and offered an opportunity to all persons involved to increase their flexibility as well as to improve their cross-cultural competencies and their mutual respect for other cultures and their respective world views (18, 19). These goals were achieved through a number of modalities: innovative electronic media, annual summer schools, workshops and seminars, international exchange of lecturers, and students who then acted as local multipliers of knowledge and experiences garnered at their universities (see below).

The collaboration between the institutes was based on equal terms. It comprised not only a unidirectional exchange between a developed country (Germany) and developing regions (Turkey, Nigeria, India), e.g., in the development and strengthening of teaching innovative methods. Of equal importance was an exchange in the other direction, e.g., in terms of the teaching of political economy of health, comparative studies in health systems, and language issues in health care delivery, where the other partners have particular expertise. The countries involved represent majority population segments belonging to the three large world religions (Hinduism, Islam, and Christianity) and numerous minority groups. The collaboration, therefore, contributed toward promoting an intercultural dialog and preventing cross-cultural conflicts (20).

### Combining Local and Distance Learning Activities

The partnership combined local and distance learning activities in order to promote collaboration in teaching and joint learning. Local activities included summer schools, workshops, and seminars, involving exchange and face-to-face contact of students and lecturers. These activities were complemented by electronic media serving as a platform for distance learning and electronic communication between students and lecturers. As is well known, distance learning has, as far back as the first half of the nineteenth century, allowed students to take part in lectures without the need to be physically present (21). In order to implement and promote distance learning facilities, the partnership used web-based communication technologies, such as a wiki-based website for communication and interaction (22, 23).

For distance learning purposes, podcasts of selected lectures given during local activities were uploaded to the website to be streamed by interested individuals unable to attend a course in person. In addition, the website served as a repository for course materials, such as publications discussed in classes and for documents produced in regular courses at all partnership institutions, such as reports written as part of student assignments. In this way, a virtual classroom was created, assisting students in experiencing how their opinions on a public health topic fare with peers in other settings who may be more directly involved with the particular problem under discussion (24). These asynchronous communication tools were well received by students and lecturers. The virtual classroom was, therefore, quite consistent with the partnership’s goal of promoting an international perspective and understanding of public health. Aside from its distance learning focus, the website facilitated communication and bridged the gap between different cultural, political, and economic backgrounds. For this purpose, it offered a set of basic social networking facilities for interested users. The technologies utilized were elements of web 2.0, a way to make use of the worldwide web through a variety of tools aiming to facilitate interaction, cooperation, and creativity (25–27). Comparable to the Wikipedia project, the website could be regarded as a dynamically and democratically formed platform cooperatively maintained and further developed by students and staff from all partnership institutions. While the tools utilized provided possibilities for synchronous (i.e., real-time) communication in the form of text and video chats, these were seldom utilized because of technical constraints and diverging course schedules.

The activities of the partnership gave opportunity to all the participants to get in touch with each other and to exchange experiences. Moreover, they brought together participants who have different backgrounds and come from countries with different economic situations and offered a direct face-to-face setting for cross-cultural understanding and respect. While, as in other disciplines also, much of the factual knowledge relevant to that field can be learned from books, (cross-cultural) social and communication skills—which are particularly important for international public health—can only be acquired through exchange with people from different countries and cultures (20).

### Initiating Structural Development through Joint Action

As far as possible, given the respective examination regulations governing modification of the curriculum, the partnership initiated structural development with regard to teaching at all institutions involved. International aspects of public health have been integrated into undergraduate, graduate, and postgraduate courses regularly taught at all universities. In addition, a procedure for recognizing course work was developed, following the idea of the European Credit Transfer System (28). The summer schools on international public health that originated at Bielefeld University have been successfully transferred to other partnership institutions. In addition, possibilities for joint tutoring of M.Sc. and Ph.D. candidates have been established, and international partners have served as examiners of the respective theses. Collaboration has also tried to work together in research resulting in joint publications (29–34).

## Challenges and Requirements for Successful Partnerships: Lessons Learned

Developing and maintaining successful academic collaborations across national boundaries can be difficult. Challenges may result from the diversity of collaborations in terms of cultural, political, geographical, and economic context. Therefore, several requirements must be met. We could learn some lessons from the International Public Health Partnership in this respect.

1. The collaboration must be based on trust, open communication, equity, and commitment. Likewise, each partner’s expectations must be clear to the others. Specific and measurable objectives of the partnerships and the responsibilities of each partner must be documented in a written memorandum of understanding. Both are necessary to evaluate the partnership activities and are key factors for a continuous quality management.
2. Regular direct or online project meetings are necessary for coordinative and planning purposes to ensure that partnership activities are not neglected and to keep all individuals involved focused at the partnership goals.
3. Partnerships that plan to establish distance learning activities need to consider the high demand with regard to IT infrastructure, including broadband internet connections, which sometimes are not reliable even in developed countries. Our experiences show that particularly distance learning activities based on real-time exchange, such as lectures simultaneously broadcast in several universities, can, therefore, quickly result in a frustrating experience. Asynchronous activities instead, e.g., implemented by means of podcasts, are more flexible and can also be easily adjusted to the specific conditions and course structures at each partnership site. Continuous IT support, e.g., for administering a partnership website, however, is indispensable.
4. Distance learning activities need to follow a low-threshold approach in order to be accepted by both lecturers and students. Also, both need to be actively included in the development of the partnership to increase motivation and commitment. In the International Public Health Partnership, students had an active role in developing and maintaining the partnership website, in organizing onsite activities, and in hosting exchange students.
5. An important aim of teaching-oriented partnerships is to initiate structural development in terms of joint degree programs and mutual acceptance of course work. Often, administrative obstacles as well as diverging regulations and structures of degree programs, e.g., regarding the length of the term or mandatory course work, can complicate this process. In addition, they can make it problematic to integrate partnership activities into regular courses. Sometimes, diverging grading systems can also make it difficult to transfer completed assignments between partnership sites (35, 36). Experiences with the International Public Health Partnership show that structural development is a time-consuming process, which also requires adjustments in existing regulations to new conditions. Therefore, sustainability of the partnership is even more necessary to render changes implemented into established structures worthwhile. Until formal agreements regarding the acceptance of course work are reached, our experiences show that, in most situations, it will be possible to decide on a case-by-case basis. It is also advisable to be as flexible as possible in recognizing coursework and examinations in order to not create additional barriers for students who plan to pursue courses abroad (37).
6. Structural development is also necessary to prevent the partnership from being dependent on the individual commitment of selected faculty members. A high staff turnover is common at institutions of higher education. Actions and outcomes of joint activities, therefore, must be thoroughly documented in order to smooth the handover of responsibilities and to ensure the continuity and sustainability of the collaboration. In this respect, also continuous institutional and administrative support is necessary. Experiences with the International Public Health Partnerships have shown that the demand in project management, e.g., in terms of accounting, can be high.
7. Each of the institutions involved has to invest a considerable amount of financial, time, and personnel resources to actively contribute to activities and to the further development of the collaboration. Otherwise, the collaboration will be hardly able to go beyond a set of intentions documented in a cooperation agreement—a situation faced by many academic partnerships (15, 35, 38). In case of the International Public Health Partnership, the partners had to contribute almost 30,000€ per year out of own resources for funding of expenses for personnel, travels, IT infrastructure, rooms, materials, accommodations, and catering not covered by DAAD. The budget situation of many universities is tight. This is also the case in Germany with resources from state general funds available for research and teaching declining for some years now (39). Financial resources of universities in low- and middle-income countries also tend to be scarce. Teaching-related partnerships with universities in developing countries, consequently, depend on continued external funding to cover expenditures for coordination, administration, infrastructure, and staff and student exchange. In case of the International Public Health Partnership, funding by the German Academic Exchange Service made it possible to cover expenses for a period of 8 years and to develop an informal cooperation between four professors into a well-functioning, successful, internationally visible formal collaboration on the institutional level. Unfortunately, as financial support ended in 2012, many of the activities of the partnership, predominantly those involving exchange of students and lecturers, can now be offered only on a restricted basis (Germany–India). This is particularly disappointing in light of the partnership’s success, the reliable structures developed, and the experiences gained. Whereas external funding agencies and foundations in most cases will only be willing to provide resources for initiating partnerships, in the long run, it must be the institutions of higher education themselves which invest resources into maintaining successful collaborations. The relevance of such collaborations for research and teaching must be acknowledged and state general funds need to be increased accordingly for this purpose. Still, expensive activities, such as transnational travels, will have to be additionally supported by other sources. In this regard, external funding agencies and foundations such as the DAAD and the European Commission (e.g., through its ERASMUS+ program) need to extend possibilities for the acquisition of complementary financial resources.

## Conclusion

As health concerns do not recognize national boundaries, the importance of international perspectives and cooperation in the teaching of public health cannot be overemphasized. By means of the International Public Health Partnership, we developed a concept for an academic collaboration that is able to meet the needs in public health education with an international perspective which also respects contextual specificities. Experiences gained with the partnership show that collaborations in the field of academic education involving mobility of students and staff face different challenges and are particularly difficult to maintain, unless sufficient financial resources are available. As the budget of many academic institutions gets increasingly tight, sustainability becomes a crucial issue. Given the relevance of international partnerships for research and teaching, more resources need to be made available for universities through general state funds to ensure long-term financial support. In addition, external funding agencies must extend possibilities for the acquisition of complementary resources.

## Author Contributions

All authors were substantially involved in the implementation and coordination of the partnership which is described in the present manuscript. PB developed the initial version of the manuscript that has subsequently been critically revised by SA, BA, KT, KN, and OR. All authors read and approved the final manuscript.

## Conflict of Interest Statement

The authors declare that the research was conducted in the absence of any commercial or financial relationships that could be construed as a potential conflict of interest.

## References

[1] Beaglehole R Bonita R Global Public Health: A New Era. Oxford: Oxford University Press (2009).

[2] Jacobsen KH Introduction to Global Health. Sudbury: Jones and Bartlett (2008).

[3] Leon DA Walt G Gilson L International perspectives on health inequalities and policy. Br Med J (2001) 322:591–4. 10.1136/bmj.322.7286.591 11238156 PMC1119787

[4] Brunner E Marmot M Social organization, stress and health. In: Marmot M Wilkinson RG, editors. Social Determinants of Health. Oxford: Oxford University Press (2006). p. 17–43.

[5] Marmot M. Social determinants of health inequalities. Lancet (2005) 365:1099–104. 10.1016/S0140-6736(05)71146-6 15781105

[6] Wilkinson RG Marmot M The Solid Facts. Copenhagen: World Health Organization (2003).

[7] World Health Organization. Closing the Gap in a Generation. Health Equity Through Action on the Social Determinants of Health. Geneva: World Health Organization (2008).

[8] Whitehead M Dahlgren G Gilson L Developing the policy response to inequities in health: a global perspective. In: Evans T Whitehead M Diderichsen F Bhuiya A Wirth M, editors. Challenging Inequities in Health: From Ethics to Action. New York: Oxford University Press (2001). p. 308–23.

[9] Crombie IK Irvine L Elliott L Wallace H Closing the Health Inequalities Gap: An International Perspective. Geneva: World Health Organization (2005).

[10] Battat R Seidman G Chadi N Chanda MY Nehme J Hulme J Global health competencies and approaches in medical education: a literature review. BMC Med Educ (2010) 10:94. 10.1186/1472-6920-10-94 21176226 PMC3019190

[11] Edwards R White M Chappel DGJ Teaching public health to medical student in the United Kingdom – are the General Medical council’s recommendations being implemented? J Public Health Med (1999) 21:150–7. 10.1093/pubmed/21.2.150 10432243

[12] Lloyd SA Byrne MM McCoy TS Faculty-perceived barriers of online education. J Online Learn Teach (2012) 8:1–12.

[13] Pfotenhauer SM Jacobs JS Pertuze JA Newman DJ Roos DT Seeding change through international university partnerships: the MIT-Portugal Program as a driver of internationalization, networking, and innovation. Higher Educ Policy (2013) 26:217–42. 10.1057/hep.2012.28

[14] Horta H Patricio MT Setting-up an international science partnership program: a case study between Portuguese and US research universities. Technol Forecast Soc Change (2016) 113:230–9. 10.1016/j.techfore.2015.07.027

[15] Etling A McGirr M Issues and procedures in forging international university partnerships. J Int Agric Ext Educ (2005) 12:15–21. 10.5191/jiaee.2005.12202

[16] Andreasen RJ Barriers to international involvement. J Int Agric Ext Educ (1997) 10:65–9.

[17] Deutscher Akademischer Austausch Dienst. Subject-Related Partnerships with Institutions of Higher Education. Bonn (2016). Available from: https://www.daad.de/hochschulen/ausschreibungen/projekte/de/11342-foerderprogramme-finden/?s=1&projektid=57300665

[18] Taras V Caprar DV Rottig D Sarala RM Zakaria N Zhao F A global classroom? Evaluating the effectiveness of global virtual collaboration as a teaching tool in management education. Acad Manag Learn Educ (2013) 12:414–43. 10.5465/amle.2012.0195

[19] Luethge DJ Raska D Greer BM O’Connor C Crossing the Atlantic: integrating cross-cultural experiences into undergraduate business courses using virtual communities technology. J Educ Business (2016) 91:219–26. 10.1080/08832323.2016.1160022

[20] UNESCO: UNESCO World Report. Investing in Cultural Diversity and Intercultural Dialogue. Paris: United Nations Educational, Scientific and Cultural Organization (2009).

[21] Moore MG Kearsley G Distance Education: A Systems View. Wadsworth: Belmont (2005).

[22] Leuf B Cunningham W The Wiki Way: Quick Collaboration on the Web. Amsterdam: Addison-Wesley (2001).

[23] Ebersbach A Glaser M Heigl R Warta A Wiki Web Collaboration. Heidelberg: Springer (2008).

[24] Duus R Cooray M Together we innovate. Cross-cultural teamwork through virtual platforms. J Marketing Educ (2014) 36:244–57. 10.1177/0273475314535783

[25] Berger P Trexler S Choosing Web 2.0 Tools for Learning and Teaching in a Digital World. Santa Barbara: Libraries Unlimited Inc (2010).

[26] Davis BG Tools for Teaching. San Francisco: Jossey-Bass (2009).

[27] Vickers R Field J Melakoski C Media culture 2020: collaborative teaching and blended learning using social media and cloud-based technologies. Contemp Educ Technol (2015) 6:62–73.

[28] European Commission. ECTS Users’ Guide. Luxembourg: Office for Official Publications of the European Communities (2009).

[29] Satish T Kannan S Sarma PS Razum O Thankappan KR Incidence of hypertension and its potentially modifiable risk factors in rural Kerala, India: a community-based cohort study. Public Health (2012) 126:25–32. 10.1016/j.puhe.2011.11.002 22133670

[30] Razum O Schaaber J Nayar KR Of silver bullets and red herrings: invited commentary to Fisk et al. Trop Med Int Health (2011) 16:669–71. 10.1111/j.1365-3156.2011.02785.x 21501348

[31] Razum O Nayar KR ’Third option’ or no option? Self-help in health care. In: Laaser U Radermacher R, editors. Financing Health Care – A Dialogue between South Eastern Europe and Germany. Lage: Jacobs Verlag (2006). p. 55–68.

[32] Nayar KR Razum O Millennium Development Goals and health: another selective development? Int Stud (2006) 43:317–22. 10.1177/002088170604300305

[33] James T Kutty VR Boyd J Brzoska P Validation of the Malayalam version of the Internalized Stigma of Mental Illness Inventory (ISMI). Asian J Psychiatry (2016) 20:22–9. 10.1016/j.ajp.2016.01.005 27025467

[34] Sathish T Kannan S Sarma PS Razum O Thrift AG Thankappan KR. A risk score to predict hypertension in primary care settings in rural India. Asia Pac J Public Health (2016) 28:26S–31S. 10.1177/1010539515604701 26354334 PMC4724234

[35] Ayoubi RM Massoud H Is it because of partners or partnerships? Int J Educ Manag (2012) 26:338–53. 10.1108/09513541211227755

[36] Arrowood RJ Hitch L The centrality of the faculty role in transnational partnerships: a research agenda. In: Blessinger P Cozza B, editors. University Partnerships for Academic Programs and Professional Development. Bingley: Emerald Group Publishing Limited (2016). p. 39–54.

[37] Halimi S Recognition: individual right and responsibility of society. In:Forum Role Conference of the Higher Education and Research Committee (CC-HER), editor. Recognition of Higher Education Qualifications: Challenges for the Next Decade. Strasbourg: Council of Europe Publishing (1996). p. 77–98.

[38] Kanter RM Collaborative advantage: the art of alliances. Harv Bus Rev (1994) 72:96–108.

[39] Marquardt W Neuere Entwicklungen der Hochschulfinanzierung in Deutschland. Köln: Geschäftsstelle des Wissenschaftsrates (2011).

